# Outcome Analysis of Distal Radius Fracture with Orthosis Versus Cast Immobilization after Palmar Plate Osteosynthesis: A Randomized Controlled Study

**DOI:** 10.3390/jpm13010130

**Published:** 2023-01-09

**Authors:** Tim Klopfer, Philipp Hemmann, Verena Rupprecht, Fabian Stuby, Ulrich Stöckle, Adrian Meder

**Affiliations:** 1 Department of Traumatology and Reconstructive Surgery, BG Trauma Center, Tuebingen, Eberhard Karls University Tuebingen, Schnarrenbergstraße 95, 72076 Tuebingen, Germany; 2 OC Bayreuth, Parsifalstrasse 5, 95445 Bayreuth, Germany; 3 Orthopedic Department, University of Tuebingen, Hoppe-Seyler-Strasse 3, 72076 Tuebingen, Germany; 4 Trauma Center Klinik am Eichert, Eichertstraße 3, 73035 Goeppingen, Germany; 5 BG Trauma Center Murnau, Professor-Kuentscher-Strasse 8, 82418 Murnau, Germany; 6 Charité Berlin, Augustenburger Platz 1, 13353 Berlin, Germany; 7 Chirurgische und Orthopädische Gemeinschaftspraxis Reutlingen, In Laisen 11, 72766 Reutlingen, Germany

**Keywords:** distal radius fracture, palmar plate osteosynthesis, hand immobilization, orthosis, circular plaster cast, patient satisfaction, Disabilities of Arm, Shoulder and Hand (DASH-Score)

## Abstract

Although the benefits of hand orthoses were shown in previous studies, they have not been able to establish themselves in clinical routines. With a focus on patient satisfaction, this study aimed to evaluate the latest generation of hand orthoses after palmar plate osteosynthesis for isolated distal radius fractures in comparison with circular plaster casts. 50 patients (16% dropout rate) were randomly assigned to an orthotic group (immobilization by orthosis, OG) or a control group (immobilization by a plaster cast, CG). Intra-articular fractures were present in 74% of the cases, and unstable AO C3 fractures in 26%. Questionnaires on patient satisfaction, documentation of the time required, clinical scores (DASH, SF-36), range of motion, grip measurements and radiographs were used for evaluation. The OG proved to be equivalent to the plaster treatment in terms of patient satisfaction, and stability of the reduction, as well as clinical scores DASH and SF-36. The OG was even superior in terms of personal hygiene (*p* = 0.011), handling (*p* = 0.008) and better adaptability (*p* = 0.013). Significantly less time was required to apply the orthosis (*p* < 0.001). In addition to the good results achieved so far, the study showed that the latest generation of orthoses has several advantages over plaster cast therapy, and could therefore become established in everyday clinical practice.

## 1. Introduction

Fracture of the distal radius (DRF) is generally one of the most common fractures, representing approximately 240 per 100,000 cases, and is therefore of major importance in everyday clinical practice. There are peak rates of fracture at 340–500 cases per 100,000 persons, in both younger (5–14 years) and older people (>50 years) [1,2]. In older patients, fractures are usually associated with osteoporosis, which complicates both the surgical treatment and follow-up [3]. Surgical therapy is the established treatment for dislocated fractures, with good results, and it has been performed increasingly often in recent years [4,5,6]. Immobilization, at least for a short time, is usually carried out both preoperatively and postoperatively.

Complications in the treatment of DRF are not uncommon, with estimated rates of 3–36% [7,8,9]. They include wound healing disorders and infections (<1% in closed fractures [9], <44% in open fractures [10]), compartment syndrome (<0.3% [11]), nerve lesions (<10% [12]), complex regional pain syndrome (CRPS; <1.4% [9]), secondary dislocation/misalignment (<1.4% [9]), tendon ruptures (<1.4% [9,13]), and pseudarthrosis (<0.2% [14]). Adequate reduction and immobilization are required for decongestion and pain reduction. Immobilization in a plaster cast or splint is initially carried out after closed reduction, as well as after surgery. The period of postoperative immobilization is determined on an individual basis depending on the severity of the fracture, bone quality, and quality of care, and is usually 0–6 weeks [15,16,17,18]. Little changed in this approach for many years. Quadlbauer et al., showed that early mobilization leads to improved functional results [19]. Occasional attempts to switch to more advanced orthoses were generally not successful despite good research results—probably due to cost differences and, above all, for reasons of manageability and practicability in everyday use [20,21]. In some cases, research studies on orthoses have even been discontinued due to problems such as secondary dislocation [22].

However, there are also limitations in classic plaster cast treatment for medical staff as well as for patients. For the medical staff, the application of a circular plaster cast is time-consuming, personnel-intensive and sometimes challenging—especially on days with high patient volume and extreme strains on staff. From a clinical point of view, a modern orthosis should therefore be easy to apply, allow uncomplicated radiographic control, and still be comparable to the gold standard cast in terms of the quality of immobilization. From the patient’s point of view, a modern orthosis should also be easy to use, allow mobilization of the free joints, and provide a better sense of hygiene. Therefore, the aim of the present study is to analyze the results of a modern orthosis compared with plaster immobilization after palmar plate osteosynthesis in distal radius fractures. For this, patient satisfaction is defined as the primary end point while retention of reduction, regaining functionality and the time required for application are defined as secondary end points.

## 2. Materials and Methods

The study was conducted in accordance with the Declaration of Helsinki and approved in the framework of “good clinical practice” by the Ethics Committee of the University of Tübingen (Project Number 173/2017BO1, 24.05.2017). The study is registered in the German Registry for Clinical Studies (Deutsches Register für klinische Studien, DRKS), number DRKS00012933. Furthermore, the study was designed according to the Consolidated Standards of Reporting Trials (CONSORT). In a monocenter, parallel group design, fifty participants were included between December 2017 and August 2019 at BG Trauma Center Tuebingen, Germany. The sample size was calculated a priori assuming an effect size of 0.9. The sample size calculation was supported by the center for clinical studies at the university of Tuebingen. Included were male and female patients between the ages of 18 and 80 years who had suffered a DRF with an AO classification 23-A2 to C3 and were scheduled for open reduction and isolated palmar plate osteosynthesis. Patients were excluded when they met one of the following criteria: (1) planned conservative therapy, (2) closed reduction not possible, (3) open fractures, (4) additional fractures in other locations and multiple injuries, (5) higher-grade soft-tissue trauma (G2 and G3 in Tscherne–Oestern classification), (6) cancer and pathological fractures, (7) initial loss of sensitivity or reduced sensation, (8) neurodegenerative diseases, including dementia, (9) wrist deformity, (10) planned surgical treatment with external fixator and/or dorsal plate osteosynthesis and, (11) lack of compliance. 

**Figure 1:**
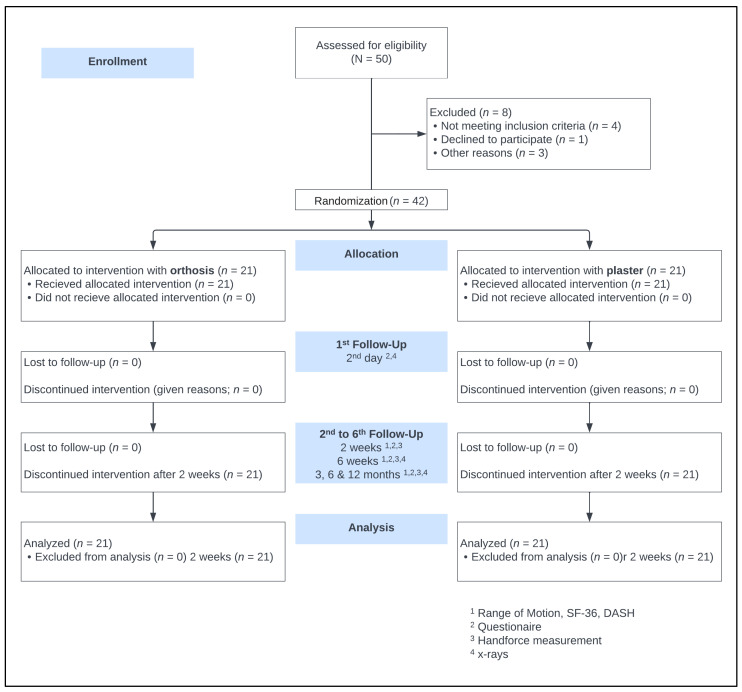
The CONSORT 2010 flow diagram.

Randomization was performed with a computer using a random number generator, labeling 50 participation certificates before the trial started. These were folded and sealed in numbered envelopes from 1–50. These envelopes were stored sequentially and numbered in a folder in the emergency department. Opening occurred after completing patient education and obtaining written consent to the study from the doctor in the emergency department. The patients were randomly assigned either to the orthosis group (OG) or the control group (CG) and were given a corresponding identification number and anonymized for the study. Since the type of splint—orthosis or plaster cast—is visible, double blinding was only possible until the time of immobilization. However, until the time of reduction and subsequent fixation, neither the staff nor the patient knew the path of immobilization. The study protocol is shown in the CONSORT flow diagram (Figure 1).

**Figure 2:**
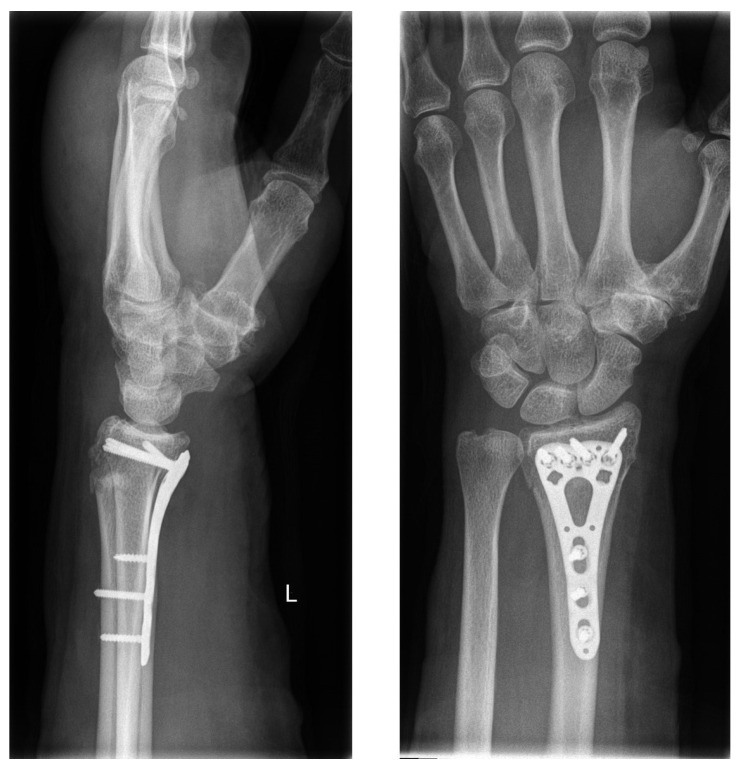
Standard operative treatment with palmar plate.

In accordance with the hospital’s standard procedure, first, the fractured forearm was lifted by finger-trap traction and was then reduced. The reduced fracture was then splinted with a circular cast or an orthosis (OPTIVOhand, OPED GmbH, Valley, Germany). After the traction was lifted, another radiograph was obtained. If an intra-articular fracture was suspected, a computed tomography (CT) scan was performed. Palmar plate osteosynthesis was performed in all patients after the swelling had subsided (VA-LCP, Synthes GmbH, Oberdorf, Switzerland; or Aptus, Medartis AG, Basel, Switzerland, Figure 2).

**Figure 3:**
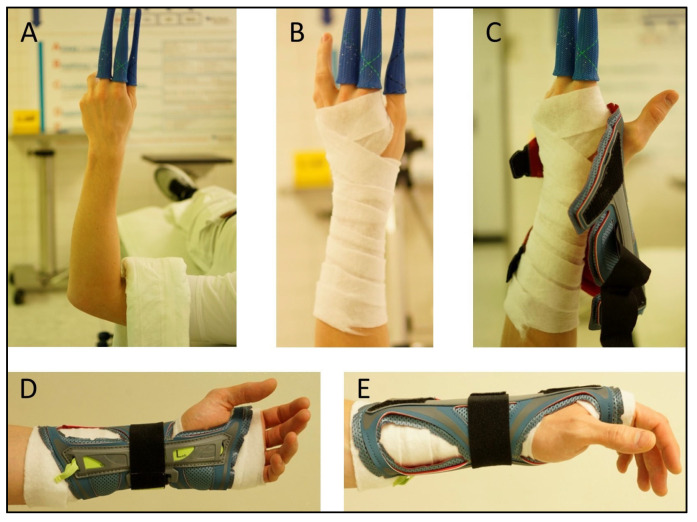
Orthosis application procedure: (**A**) Hanging in extension, (**B**) Cotton-wool padding, (**C**) Placement of the orthosis in suspension, (**D**,**E**) Correct placement of the orthosis.

Postoperatively, immobilization was carried out with a dorsal plaster splint versus the OPTIVOhand orthosis for 2 weeks, with mobilization of the adjacent joints. The used orthosis consists of a flexible polyester textile fabric with flexible adjustable Velcro fasteners and is reinforced by aluminum splints. It weighs 600–800 g. It is washable and can be used with an interchangeable splint for either the left or right wrist. The two available sizes were selected depending on the circumference of the patient’s wrist. In both plaster casting and orthotic therapy, a thin layer of cotton wool padding was applied to avoid pressure points caused by swelling (Figure 3A–E).

The time required to apply the orthosis and the cast was measured during the initial application. In view of the several variables capable of affecting the time measurements, only the placement of the circular white plaster after finger-trap traction was measured using a stopwatch. The time required for hardening, splitting, and checking was excluded from the measuring. Casts and orthoses were both applied in the emergency room for primary care as well as in the operating room after surgery under the supervision of the medical staff; any problems were recorded using a questionnaire. Follow-up examinations were performed in accordance with the scheme after 2 days, 2 weeks, 6 weeks, and 3, 6, and 12 months (Figure 1). The sutures and immobilization devices were removed 14 days postoperatively. For the follow-up, patients completed the disabilities of the arm, shoulder and hand questionnaire (DASH), and the Short Form-36 questionnaire (SF-36) including physical (PCS) and mental component summary (MCS). A questionnaire on patient satisfaction with the splint used was also given to both groups. This questionnaire included the items handling, hygiene, the accuracy of fit, pressure complaints, aesthetics, and dressing on a 5-point Likert scale. Additional to the ROM and grip strength assessment, surgeons completed a questionnaire about fit accuracy, signs of secondary dislocation, ease of application and pressure points. Furthermore, radiographic imaging was carried out on two planes.

Statistical analysis was performed using IBM SPSS Statistics for Windows, version 25 (IBM Corporation, Armonk, New York, NY, USA). The Kolmogorov–Smirnov test was used to test the distribution of the groups, with a value of *p* < 0.05 indicating no significant differences in the distribution. Chi-square tests, for categorical variables, and t-tests, for interval-scaled variables, were used to test if the group characteristics differed initially. Furthermore, a repeated measures analysis of variance (ANOVA) was used to examine differences between the study groups over the entire course of treatment and follow up. If the assumption of sphericity was violated, tested by Mauchly’s Test, the results were corrected by the Greenhouse–Geisser method. To avoid the error accumulation by multiple testing, the Bonferroni-Holm method was used in post hoc analysis. The significance level was set at *p* < 0.05.

## 3. Results

A total of 50 patients with isolated DRFs were enrolled in the study between December 2017 and August 2019. The dropout rate was eight patients (16%). Follow-up data were completed for 21 patients in each study group. The most common reason for dropouts was a change in the treatment regimen after inclusion in the study, such as a decision to use an external fixator due to increased instability (*n* = 3) or additional dorsal plate osteosynthesis (*n* = 1). Table 1 shows the patients’ age and sex distribution. The right arm was affected more often in both groups, at 62% in the CG and 57% in the OG (*p* = 0.753). In both groups, there was a predominance of severe C-type fractures in the AO classification, affecting a total of 31 patients (74%). There were no significant differences in the distribution of AO classifications (*p* = 0.520).

### 3.1. Study-Specific Questionnaire

There were no significant differences in levels of patient satisfaction regarding general physical condition, except for physical capacity 6 weeks postoperatively. Patients in the OG felt significantly more restricted than those in the CG (*p* = 0.042). However, the OG patients were significantly more satisfied with the splint’s accuracy of fit (*p* = 0.007). There were no significant differences between the two groups in relation to wrist pain during the postoperative follow-up examinations. There were no significant differences between the groups in relation to exercise capacity at 2 weeks postoperatively (*p* = 0.469). Patients in the CG could do slightly more exercise after 6 weeks (*p* = 0.042). However, this difference was no longer evident 3 months postoperatively (*p* = 0.458). The handling of the splint was rated as significantly easier (*p* = 0.008). The patients in the OG felt significantly less restricted in personal hygiene (*p* = 0.011). There was a significant difference in the patients’ assessment of subjective adaptability in favor of the OG (*p* = 0.013). No significant differences were noted in relation to pressure symptoms, disturbance of appearance, or difficulties in dressing.

### 3.2. SF-36

**Figure 4:**
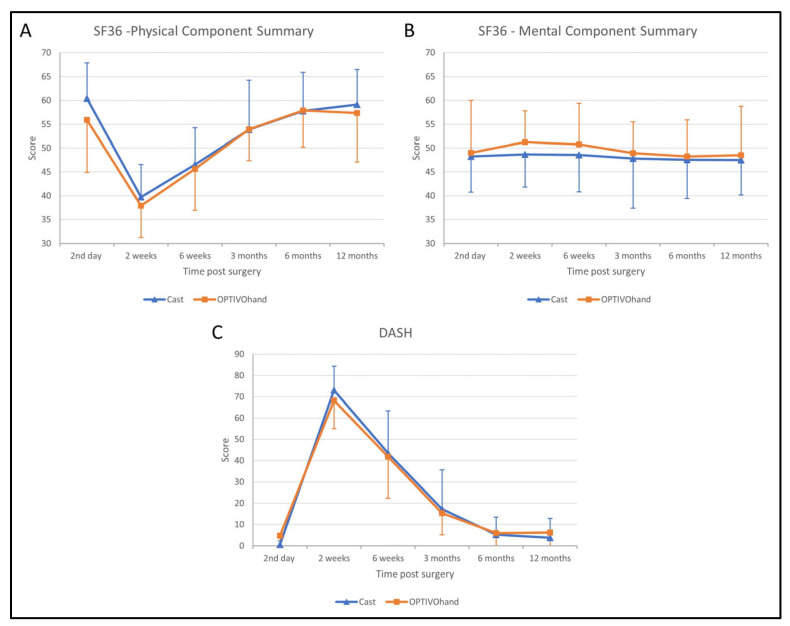
Patient outcome scores during follow-up ((**A**) SF36—Physical Component Summary; (**B**) SF36—Mental Component Summary, (**C**) DASH). Blue triangles represent immobilization by circular cast, orange squares represent immobilization by OPTIVOhand orthosis.

Statistical analysis of the PCS score of the SF-36 revealed a significant effect on time after surgery (F (3.680, 139.842) = 71.481, *p* < 0.001). However, there was no significant difference between both groups during the whole follow-up (F (1, 38) = 0.547, *p* = 0.464). In both groups, the PCS scores significantly declined between the second day after surgery to a minimum at 2 weeks follow-up and recovered until 6 months follow-up. Further, there was no statistically significant interaction effect between time after surgery and the study groups (F (3.680, 139.842), *p* = 0.522; Figure 4A). 

Similar to the PCS score, the MCS score differs significantly over time (F (3.299, 125.354) = 45.070, *p* < 0.001). Hereby, the MCS score was significantly increased over 2 to 6 weeks post-surgery. The significant effect between both groups (F (1, 38) = 5.345, *p* = 0.026) was not confirmed in the post hoc *t*-test due to Bonferroni-Holm correction. There was no significant interaction between the group and time after surgery (Figure 4B).

### 3.3. DASH

The significant effect over time was also confirmed on the DASH score (F (3.204, 124.940) = 255.468, *p* < 0.001). Both groups significantly improved 2 weeks after surgery and recovered after 6 months (Figure 4C). There was neither a significant effect on the DASH score between both groups over the whole follow-up (F (1, 39) = 0.004, *p* = 0.948) nor an interaction effect between the type of orthosis and follow-up (F (3.204, 124.940) = 0.906, *p* = 0.445).

### 3.4. Maintenance of Reduction

To determine the loss of reduction, the reduction was controlled during finger-trap tracking before immobilization and after the application of cast or orthosis application without traction. Thus, the preservation of reduction was verified twice consecutively by two radiographs or one radiograph and one CT scan. Loss of reduction was defined as a loss of palmar inclination of >10°. This occurred seven times in the OG (14%) and 10 times in the CG (20%). There were no statistically significant correlations between the groups and the occurrence of loss of reduction (*p* = 0.412).

### 3.5. Application Time

The OG clearly showed a significant advantage in terms of the time required for the application: the plaster application took an average of 7 min 30 s (SD 2 min 32 s), while the orthosis only took 2 min 5 s (SD 1 min 38 s), *p* < 0.001. Not included was the time for hardening and splitting of the plaster.

### 3.6. Clinical Examination

**Figure 5:**
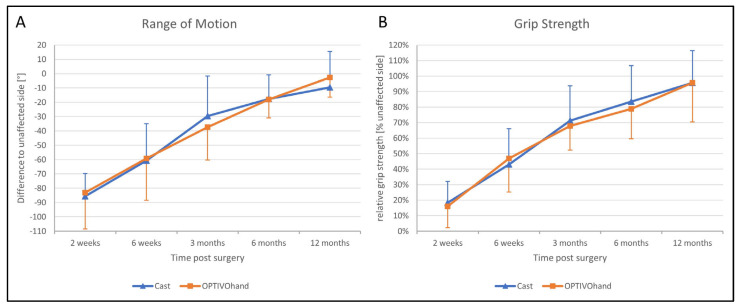
Functional outcome ((**A**) range of motion; (**B**) grip strength) after 2 weeks to 12 months follow-up.

The functional outcome 2 weeks postoperatively after removal of the splints was still limited in comparison with the healthy hand. In both groups, patients were able to significantly improve their ROM (with extension and flexion added, F (2.844, 79.623) = 96.840, *p* < 0.001; Figure 5A) and grip strength (as a percentage of the strength on the healthy side, F (2.679, 61.628) = 112.577, *p* < 0.001; Figure 5B) over the entire study period. Independently of the type of splint (ROM: F (1, 28) = 0.011, *p* = 0.918), patients achieved almost comparable mobility and strength of the healthy side after 1 year. The total ROM 2 weeks postoperatively was limited by a mean of 66° (SD 6°), whereas after 1 year the deficit was only 9° (SD 3°). With no sig. differences between both groups (F (1, 23) = 0.044, *p* = 0.836), all patients increased the relative grip strength from initially 17% 2 weeks after surgery to 96% after 1 year on average. There were no significant differences between the OG and CG in relation to mean circumference differences, as a measure of swelling. 

### 3.7. Surgeon Questionnaire

The surgeons did not observe any significant differences in the assessment of fit accuracy, signs of secondary dislocation, or ease of application and pressure points.

### 3.8. Complications

A total of four complications occurred that required surgical treatment, three in the CG (14%; (1) secondary dislocation of the dorsal fracture zone after palmar plate osteosynthesis, (2) Secondary dislocation with consecutive shortening of the distal radius (3) B3 fracture “Reversed-Barton-Fracture”) and one in the OG (5%; rupture of the extensor policis longus tendon). None of these complications were caused by the use of the orthosis or the plaster cast. All of the complications were successfully treated with an appropriate revision operation.

## 4. Discussion

This study shows that using a prefabricated orthosis for the immobilization of DRFs before and after surgery is equivalent to the standard treatment with plaster casting. Aspects such as perceived personal hygiene, handling, and adaptability were rated better by patients of the OG. One reason why orthoses might be integrated more into everyday clinical work is the fairly small amount of time and expertise that is required to apply and adjust it to the patient. The DRF is still one of the most common types of fracture in general. Even today, the largest age peak is seen in 75–84-year-old patients [2], and a significant increase in these numbers is expected because of demographic trends [23]. As they are usually a result of osteoporosis, these fractures are challenging both for surgical procedures and for follow-up treatment [3]. In younger individuals, early or even immediate mobilization may seem reasonable and may be associated with better functional outcomes [24,25]. However, other studies have shown that initial immobilization also provides benefits. For example, it has been shown that the need for analgesics, especially opioids, can be reduced [26]. The previous gold standard, plaster casting, has not yet been replaced in everyday clinical work by any of the manufactured orthosis models, that have received some good research results. Stuby et al., were already able to demonstrate very good results with a similar orthosis in relation to ROM, SF-36, and DASH in 2015 [20]. One reason for orthoses being rarely used in everyday clinical work may have been their size and the more difficult adjustment to the limb when there is a lack of practice. Switching from plaster casting to orthoses postoperatively may then be impracticable for various reasons. The aim of the present study was therefore to further eliminate the above-mentioned problems to demonstrate that by using a suitable orthosis improved care can be achieved.

**Figure 6:**
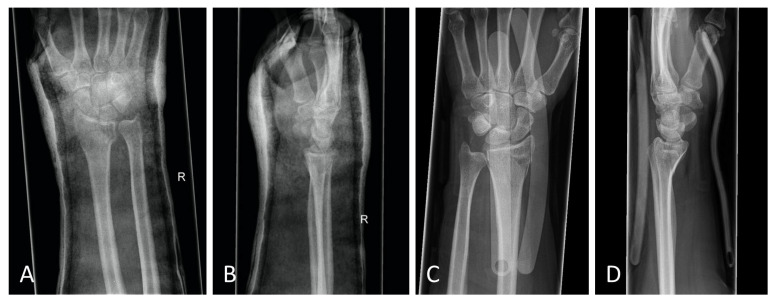
(**A**) Posteroanterior radiograph with cast. (**B**) Mediolateral radiograph with cast. (**C**) Posteroanterior radiograph with orthosis. (**D**) Mediolateral radiograph with orthosis. All radiographs were taken after reduction before surgery.

**Figure 7:**
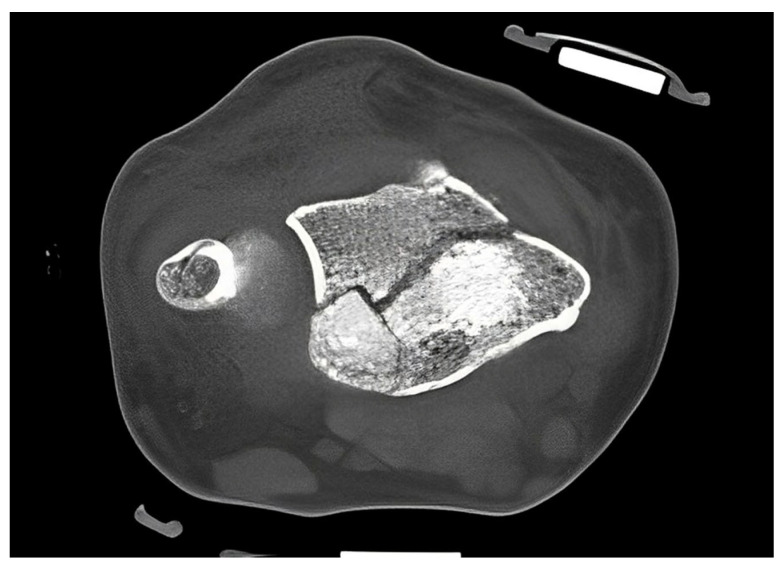
CT scan of a DRF in transversal view. The use of aluminum-based orthosis does not induce metal artifacts.

The standard treatment approach in our Level I trauma center was not altered by the study protocol, except from the placement of the orthosis. In our approach, we were able to adjust the orthosis used immediately during finger-trap traction and show that there was no increase in secondary dislocations on the radiographic and CT control imaging and, moreover, that diagnostic procedures were in no way restricted when the orthosis was in place. The quality of detail was not limited in any way in the lateral projection, very less in the posteroanterior view (Figure 6A–D). Additionally, the use of the orthosis did not affect the CT scan. As shown in Figure 7, there are no metal artifacts induced by the aluminum splints. Hence, modern orthoses enable comprehensive radiographic diagnostics. 

Even in unstable AO type C3 fractures, the orthosis achieved very good stability and acceptance. No increased dislocation in the orthosis was observed. A significant time saving was also noted, as well as easy handling during placement. A clear advantage in comparison with previous studies is the fact that the orthosis remained with the patient from the first day of trauma care and could be used and adjusted at any time without problems. As the orthoses have a slim and lightweight design, significantly better personal hygiene, adjustability, and handling were observed. In the regular postoperative follow-up visits, no other significant differences were observed in relation to loss of reduction (>10%), DASH score, SF-36 scores, or grip strength, apart from better mental health component responses.

After the removal of the orthoses and plaster splints 2 weeks postoperatively, both study groups received accompanying physiotherapy to maintain or regain mobility and strength. Although various studies have shown that early mobilization can also achieve early mobility, the authors of this study believe that immobilization for 2 weeks is advisable, especially in the case of initially very unstable fractures and concomitant osteoporosis. This does not entail any significantly increased risk of persistent movement restriction [25,27,28]. If mobilization is permitted during a physiotherapy session, the orthosis can be removed and reapplied in an easy and fast manner.

A meta-analysis by van Delft et al. recommended in 2019 that immobilization should not last longer than 3 weeks, if possible [16]. In exceptional cases, prolonged immobilization may also have to be accepted. This is possible without any problems with the orthosis used in this study, as it can be easily adjusted and washed to maintain hygiene. On very hot and humid days, the orthosis has clear advantages over the standard plaster cast in terms of stability and hygiene of the material. In contrast to a circular cast, orthoses allow preoperative and postoperative wound checking due to their integrated openings. It can also be adjusted in case of increasing or decreasing swelling. Particularly in busy emergency rooms or when there are staff shortages, the simple and quick application of orthosis can save a lot of time and stress. These orthoses are not recommended in patients with dementia or lack of cooperation, as they can be removed too easily in comparison with a circular cast. The problems reported in previous studies and orthoses have been analyzed and consistently improved throughout the latest device generations. The clinical results in the present study are sufficiently persuasive, so further studies on conservative therapy, especially in geriatric patients, would be desirable.

The study has a few limitations: the number of patients is small and dropouts occurred. These were mainly due to the rigorous inclusion and exclusion criteria. Patients were excluded if procedures were changed during the decision-making process; this occurred more often than expected. Only palmar plate osteosyntheses were included, in order to ensure sufficient comparability.

## 5. Conclusions

The results of this study indicate that both preoperative and postoperative reduction can be maintained with an orthosis and that there are no disadvantages in comparison with the current gold standard of plaster casting. The orthosis allows quick and easy application, opportunities for adjustment during therapy, better personal hygiene and can reduce the workload of hospital staff.

## Figures and Tables

**Table 1:** Subject characteristics. Independent *t*-test (age) and Chi-square test (sex, operated side, type of fractures) were used to test if both groups—OG and CG—differed initially.

	Total (*n* = 42)	Orthosis Group (*n* = 21)	Control Group (*n* = 21)	*p*
**Age in years (± SD)**	57 (14)	58 (12)	56 (17)	0.793
**Female sex (*n*, %)**	32 (76 %)	17 (81 %)	15 (71 %)	0.469
**Operated side right (*n*, %)**	25 (60 %)	12 (57 %)	13 (62 %)	0.753
**Type A fractures**				0.520
A2	2 (5%)	0 (0%)	2 (10%)	
A3	7 (17%)	5 (24%)	2 (10%)	
**Type B fractures**				
B3	2 (5%)	1 (5%)	1 (5%)	
**Type C fractures**				
C1	8 (19%)	3 (14%)	5 (24%)	
C2	12 (29%)	7 (33%)	5 (24%)	
C3	11 (26%)	5 (24%)	6 (29%)	

## Data Availability

All data, tables and figures presented in this manuscript are original. Further inquiries can be directed to the corresponding author.
